# Vision Restoration in Glaucoma by Activating Residual Vision with a Holistic, Clinical Approach: A Review

**DOI:** 10.5005/jp-journals-10028-1237

**Published:** 2018-03-01

**Authors:** Bernhard A Sabel, Lizbeth Cárdenas-Morales, Ying Gao

**Affiliations:** 1 Professor, SAVIR Center, Magdeburg, Germany; Institute for Medical Psychology, Otto von Guericke University of Magdeburg Magdeburg, Germany; 2 Lecturer, Institute for Medical Psychology, Otto von Guericke University of Magdeburg, Magdeburg, Germany; 3 Researcher, SAVIR Center, Magdeburg, Germany; Institute for Medical Psychology, Otto von Guericke University of Magdeburg Magdeburg, Germany

## Abstract

**How to cite this article: **Sabel BA, Cárdenas-Morales L, Gao Y. Vision Restoration in Glaucoma by activating Residual Vision with a Holistic, Clinical Approach: A Review. J Curr Glaucoma Pract 2018;12(1):1-9.

## THE NEED FOR A HOLISTIC APPROACH TO EYE HEALTH

People with visual system problems go to the ophthalmologist. If the problem is with the optical apparatus of the eye, the cornea or the lens, prescribing a pair of glasses or surgery to replace a clouded lens is an effective solution, which may give instantaneous improvement in visual performance, and may feel like a miracle.

But if the diseases attacks the nervous system part of the eye, such as the retina, optic nerve or the brain, the case is more difficult. These structures are responsible for transmitting and processing visual signals from the outside world to the brain, the place where visual experience occurs. In this case, ophthalmology has limited possibilities to offer help, because the traditional ophthal-mological view holds that once the damage has happened, the vision loss is mostly irreversible. This pessimistic and often frightful message is passed on to patients who suffer diseases, such as glaucoma, diabetic retinopathy, optic neuropathy, optic nerve damage, amblyopia, stroke, or brain trauma. The best one could hope for is that the therapy can stop the progression of the disease and patients can learn to cope.^1^ An improvement is not generally expected, because of the dogma: “blind stays blind.”

We should not be satisfied with this pessimistic dogma because when the nervous system is affected in other diseases of the brain, it exhibits enormous capacities to adapt to the damage.^2-4^ Since one of the brain’s main tasks is to learn (and the brain does this every day), this remarkable adaptability to change, called “neuroplas-ticity,” gives the brain a special place among all other organs.^5^ Neuroplasticity is the fundamental mechanism of neurological rehabilitation of motor function, speech, sense of touch, memory, and cognition. Yet, the traditional and sticky dogma claims that the visual system does not benefit from any plasticity potential and hence, cannot recover from damage. As we discuss now, this skeptical view is becoming outdated and there is indeed more hope. Even in normal vision, and also in so-called *eye *diseases, the brain has an important role to play, even in being able to compensate for damage.

If we ask ourselves how important the brain is for normal vision, we could ask the simple question as to how many grams of nerve tissue of the human body is involved in visual processing and where that tissue is located. The answer is surprising: about 1 gm of nerve tissue is located in the eye (retina) but several hundred grams are found in the brain. Yet, if we have a vision problem, we go only to the “eye” doctor who thinks about that 1 gm and we do not visit any expert taking care of the rest. The clever patient may then come up with the idea of consulting a neurologist, who is more familiar with the brain. Neurologists are responsible for nervous system and brain disorders, such as Parkinson’s disease, stroke, or dementia. But if patients wish to learn more about their visual problem, they will usually be sent back to the ophthalmologist, who is responsible for treating “vision” problems. Then patients may feel like sitting between two chairs and no one can really help. And there is another problem: visually impaired patients are understandably scared, worried, or even depressed as a dramatic change of life is often feared. Therefore, other disciplines are needed, such as psychiatry or psychology, which have very little to do with visual impairments. This can be very frustrating and the patient feels completely abandonned in this situation. Visually impaired people have little chance to get their visual problem examined or treated with a “holistic” perspective. Therefore, it is not enough to just look at the eyes, ignoring the brain and the person behind the eyes.

Visual impairments cause suffering, and help seems “out of sight.” But the prospects are improving. Thanks to the research progress in the field of vision restoration, we have learned much about brain plasticity during the last 30 years. This article summarizes the scientific basis of a holistic, clinical treatment approach how residual vision can be reactivated and vision improved or restored. The depressing dogma “blind stays blind” is crumbling and there is some light at the end of the tunnel.

## A MICROPHONE—ONLY TOGETHER WITH AN AMPLIFIER—CAN RECORD MUSIC

To understand the mechanisms of vision recovery, we first need to look at how “normal” vision works, a complex process. In addition to the healthy functioning of the optics and the retina in the eye, a well-functioning brain is critical. One can think of the eye as a “microphone” that transforms not sound but light rays into electrical (neuronal) impulses. These are then sent via the optic nerve to the brain, where the signals are analyzed and interpreted, creating the subjective sense of a visual experience. Like the circuit boards in a music amplifier, the brain also has many electrical (neuronal) circuits which are extremely complex. Vision is thus not only a product of the eyes, but above all, the result of complex information processing in the brain.

Therefore, when the retina is damaged, we should also look at the brain and its untapped capabilities, and ask whether the reduced information flow can be improved by means of neuroplasticity to “amplify” the signals. To use an analogy: if you walk down the stairs into a dark basement which is illuminated only by a weak light bulb, you cannot see anything until the dark adaptation takes place. In a similar manner, if less information from the eye reaches the brain, the brain amplifier must adapt to maximize the reduced amount of information.

This circumstance is taken into account by a “holistic” treatment of visual impairment: not only the function of the eye must be optimized, but also that of the brain. The goal is therefore, not only to regulate eye pressure or retinal cell survival, which is what ophthalmologists do with eye drops, or surgery, but also to optimize the brain to maximize the use of residual visual inputs. Thus, while traditional ophthalmology aims to slow down or even prevent the aggravation of the problem (cell death), the new holistic, clinical approach of vision therapy is to strengthen residual vision, by optimizing the function of the remaining cells and brain networks to strengthen their maximum potential of the remaining structures.

## THE “EYE-BRAIN-VASCULAR” TRIAD

The fastest and cheapest way to improve your vision is to optimize the (physical) visual stimuli themselves so that more light can enter the eye. Simply use stronger light (e.g., use 150 W bulbs in your home) to improve contrast, and magnify the letters, etc. A holistic treatment optimizes residual vision by improving eye, brain, and blood flow. The “eye-brain-vascular” (EBV) triad ensures that all the processes required for good vision work together well and in a balanced manner. As Table 1 summarizes, there are many interactions among these three elements and visual problems can arise, if any one of them is disturbed. This complex interaction among the eye, brain, and vascular system has both disadvantages and advantages. The bad news is that there are many causes that can lead to visual problems. But there is also good news: these are possible mechanisms that can improve low vision. This explains why so many different “alternative methods” may be useful in the treatment of visual impairments, even though their mechanisms are perhaps still poorly understood or have not yet even been studied scientifically.

**Table 1:** **Table 1: **Elements of normal visual functioning and their interactions. For brevity, the term “Eye” implies also “Vision.” Abnormalities in any one of these functions can lead to imbalances and compromise healthy vision

Brain-Eye-Vascular Interactions			
**E2B**		**B2V**	
Phototransduction		Autonomic innervation	
Neural processing and transmission		Acute and chronic stress	
Melatonin/circadian rhythms		Stress hormones	
Thermoregulation		Endothelin, cortisol	
		Pressure regulation	
**B2E**			
Neural processing		**V2B**	
Eye and head muscle control		Blood supply	
Iris contraction		Intracranial pressure	
Intracranial pressure		regulation	
Visual analysis/		Microsaccade-heartbeat	
interpretation		coupling	
• Vigilance			
• Focal/global attention		**E2V**	
• Visual memory		IOP	
• Motivation		Perfusion pressure	
• Expectation/cognition			
• Emotions/stress		**V2E**	
• Stressors		Blood supply	
• Atmospheric pressure		Vascular dysregulation	

E: Eye and vision; B: Brain; V: Vascularity

In our holistic vision therapy, we therefore pursue various scientifically sound approaches with the goal to strengthen residual vision^6^:

- Reactivation of “silent cells” and neural transmission from the eyes to the brain,
- Optimization of impulse processing in the visual structures in the brain, and
- Influencing all those functions that either directly or indirectly affect visual processing (e.g., attention, eye movements, cognitive processing, and stress).

The goal to optimize residual vision can be achieved in two ways: (i) by improving blood circulation and (ii) by enhancing synaptic transmission between neurons. The EBV triad shows how closely the eye, brain, and cardiovascular system work together to foster good vision. The eye should not be considered in isolation, but must be considered in the context of the brain and vascularity in order to maximize the reactivation of residual vision.

### Reactivating “Silent” Cells and Neurovascular Coupling

As animal experiments show, there are not only healthy and dead cells in the damaged visual system, but also many diseased, hypometabolic nerve cells. They survive but become inactive as if being locked in a hibernation mode. It is possible to reactivate these “silent neurons” with approaches that fundamentally differ from the methods of conventional medicine. While conventional medicine seeks to save cells from death to stop the progression of the disease, our path is to reactivate living but silent cells to function again.

To understand how nerve cells can be reactivated, let us make a small excursion into the science of “neuro-vascular coupling”. It explains the interaction of blood supply and nerve cells activity, both of which are closely coupled with another. Specifically, if nerve cells want to fire electrical signals from the eye to the brain (or within the brain), they need sufficient energy, i.e., oxygen and glucose. Nerve cells in an idling mode consume little oxygen, but while firing action potentials, there is a need for considerably more oxygen when, for example, light flashes stimulate the photoreceptors of the retina and more oxygen is required for processing the neural impulses triggered by the photoreceptor activation. Since neuronal activity leads to the release of potassium ions as the impulses (action potentials) propagate along the axons toward the brain, tiny blood vessels (microcircula-tion) near the cells respond to the potassium increase by dilating to increase blood flow. Since such ionic flow is a microelectric current signal traveling upstream along the blood vessel wall, this triggers the vascular system to dilate so as to enhance glucose and oxygen delivery downstream and to support the activated neurons. The increased oxygen now enables the cells to fire their signals. But when blood vessels do not respond properly to this signal because of “vascular dysregulation”, the neurons, deprived of oxygen like a car without gas, stay silent. Too healthy to die, but not healthy enough to work.

### Vascular Dysregulation

Neurons can become silent because they are either molecularly damaged, inhibited by toxic substances, or lack of oxygen supply. They now remain inactive like a car without gasoline and will not fire action potentials, although—in principle—they could. One major cause of their “silence” could be “vascular dysregulation,” a field of research that was pioneered by Prof Flammer from Basel, Switzerland.^7-10^ Here, the vascular blood supply is compromised for several possible reasons, which can occur individually or in combination: (i) increased intraocular pressure (IOP) can strangulate capillaries partially or completely, (ii) blood flow is generally reduced when, e.g., blood pressure is too low, (iii) blood vessels in the eye or brain are suddenly constricted (cramped) due to an acute stress event (“spasm”) or are occluded by a blood clot, (iv) the difference between arterial blood flowing to the eye, brain, and venous blood drain is too low, (v) the oxygen content in the blood is too low (in smokers or mountaineers at high altitudes above 2,000 m), or (vi) vascular dysregulation develops due to continuously increased levels of stress hormones in the blood due to prolonged stress. Other causes may be unhealthy lifestyles (e.g., smoking, alcohol, obesity, unbalanced nutrition) or uncoordinated control of the blood vessel walls by the nervous system, so the oxygen supply does not match the need of nerve cell activity in the retina or brain. Many of these factors are controlled or modulated by the brain and are closely related to our general way of life, e.g., our own behavior in combination with some genetic susceptibility cofactors. The well-coordinated EBV triad, i.e., the healthy interaction among the eye, brain, and blood supply, is therefore, a critical biological basis of healthy vision. Any disruption in this triad may lead to visual impairment.

### Visual Processing in the Brain

Besides the important blood supply, it is the processing by neuronal networks in the brain that matters most in healthy vision. Let us assume that, e.g., only half of the cells in the retina and optic tract are still alive after an optic nerve damage. Now the key question is: how many cells have actually died and how many are still alive but only “functionally inactive,” like in a hibernation mode? When all the cells are dead, there is, of course, no chance of vision recovery; but if inactive cells exist at all, we should try our best to reactivate them. There are many direct and indirect processes and functions within the brain that are needed for healthy (normal) vision. These can be recruited to “wake up” the silent neurons and thus optimize signal processing in the brain. Direct factors include focal and global attention, expectations (positive attitudes), fatigue, acute and chronic stress, emotions, depression and microsaccades (miniature eye movements important for high-resolution vision/visual acuity). Indirect factors include atmospheric pressure (weather sensitivity), which can alter blood and intracranial pressure, time of day, and circadian rhythms.

In conclusion, many factors of the nervous system and of the blood vascular system have a direct or indirect effect on vision. So, besides cell survival, the interplay of “vascular” and “neural” mechanisms also contributes to vision problems. While all these factors are part of the problem, the good news is that they are also part of the solution.

## A HOLISTIC APPROACH TO VISION RESTORATION

Holistic vision therapy means that all relevant factors are considered for individual and personalized patient care: a well-balanced EBV triad and optimized neural processing in the brain. The term “holistic medicine” should not be confused with the term “alternative medicine” because the holistic approach complements—not replaces—existing and proven methods of conventional medicine. Though often referred to as being “esoteric practices,” the terms “holistic” and “alternative” medicine are not magical in any way and often (but not always!) have a rational explanation. Yet, holistic therapy focuses on methods that can be explained based on scientific evidence and/or sound scientific arguments. We need to be open to such new solutions and inform our patients in detail why these procedures are selected for the individual treatment. We should not focus only on a single mechanism of action (e.g., only on IOP), but we should combine different therapy modules to maximize residual vision and augment a patient’s individual psychological resources. This is the core of our holistic, clinical SAVIR therapy approaches (www.savir-center.com).

### Which Eye Diseases are Treatable with Holistic Vision Therapy?

In contrast to conventional medical methods, which often rely on a single mechanism of action, the holistic therapy has a comprehensive toolbox available that simultaneously addresses a wide variety of mechanisms. In addition, the brain does not care why the visual damage has occurred. The brain is interested in how residual (unused) potential can best be utilized. That is why the holistic vision therapy is suitable for a wide range of disease including glaucoma, optic neuropathy (inflammatory, traumatic, or unexplained), diabetic and vascular retinopathy, age-related macular degeneration, amblyopia, and posttumor or infection status. Diseases of the brain, such as stroke or brain trauma, can also be treated; however, their recovery prognosis is lower than that of eye or optic nerve diseases. The holistic therapy aims at people who still have residual vision. It is not recommended to the completely blind and it is not used to treat diseases of the optical apparatus of the eye (cornea, lens), e.g., short- or long-sightedness, or cataract.

### Elements of the Holistic Vision Therapy

Transorbital Alternating Current Stimulation

**Figs 1A and B:**
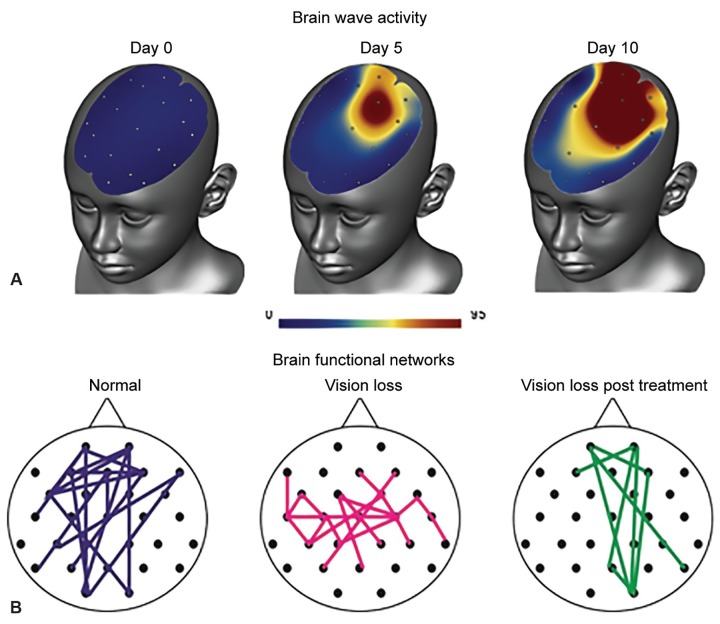
Effects of ACS treatment as shown by brain wave activity (upper panel) and normalized functional networks in the alpha-band (8-12 Hz)

**Figs 2A and B:**
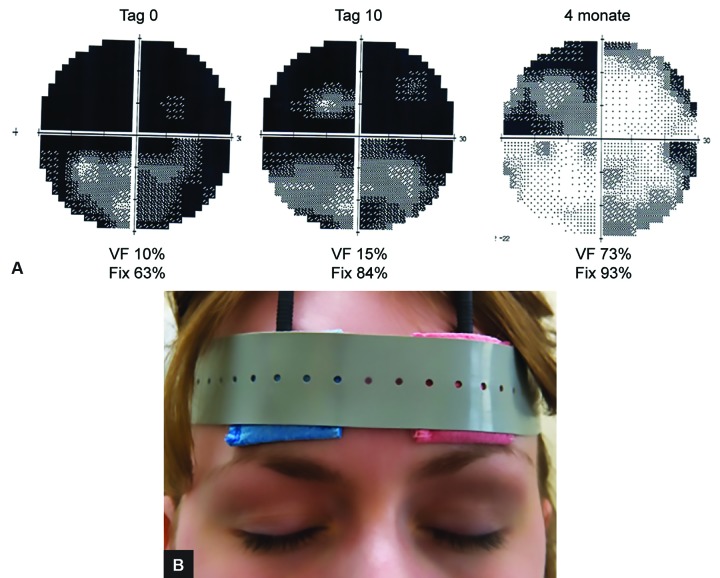
Visual field of 27-year-old Thomas P, 1 year after optic nerve damage. Treatment: 10 days of alternating current stimulation, followed by 4 months of eye yoga, antistress program, and sports. He has a positive and optimistic personality, is socially and professionally integrated. VF: Visual field; Fix: fixation ability (stability of the eyes); Lower panel: position of the stimulation electrodes

Daily treatment with alternating current stimulation (ACS) is our core therapy (Figs 1 and 2). This treatment entails the delivery of microcurrent pulses through a “brain pacemaker”, a most effective way to improve residual vision. The treatment is based on more than 15 years of research in our laboratory at the Otto von Guericke University in Magdeburg, where we treat mostly people with damaged optic nerves and glaucoma. The ACS pulses stimulate all nerve cells of the retina and force them to simultaneously fire while, at the same time, improve blood circulation. Recent clinical studies on various visual system disorders by different groups have shown that electrical stimulation can improve vision even many years after injury.^11-20^

Treatment with the brain pacemaker is noninvasive, painless, and it uses neither drugs nor surgery. It is performed daily for 30 to 50 minutes for ten days. Patients sit on a chair and with electrodes attached to the forehead, through which very weak microcurrent pulses are administered (Fig. 2). This forces the nerve cells in the retina to fire rhythmically. The electrical stimulation gives the patient the sense of faint, pulsating lights, so-called phosphenes. Adverse effects are minimal (mild tingling during treatment, rarely temporary headache); not a single serious adverse event was ever noted.

The firing of nerve impulses target the eye and brain, which leads to improved blood flow because of cardiovascular coupling. This not only helps neurons to fire, but also wakes up those “silent” cells in their neighborhood. Another effect of ACS is a normalization of brainwave patterns by reorganizing neuronal communication networks across the entire brain (Fig. 1). Figure 2 shows the electrodes placed on the forehead and the following YouTube video demonstrates how the treatment works: www.youtube.com/watch?v=g8p3mWsLvAI.

Scientific studies and our experience in the clinic have shown that about 70% of patients notice improvements in their visual functions, subjectively or objectively, with average improvements of about 24% of the whole visual field and 60% of the damaged area.^15^ These improvements are usually stable for at least 6 to 12 months. In some patients, they are maintained for years (often depending on how well the patient continues the home exercises). Physiologically, this treatment results in an improvement in brainwave activity that shows both more “good” alpha waves and improved connections in the brain network.

Eye Yoga

Yoga is a collection of physical and spiritual exercises from ancient India which includes certain postures (“asanas”) for strengthening and relaxing the body as well as breathing exercises (“pranayama”). Just as in yoga, where the muscles of the whole body are systematically contracted and stretched, eye yoga trains eye muscles. Here, the eyes are systematically moved into all directions: up-down, right-left, in a circle, in a figure-of-eight, and directed toward the tip of the nose. This is flanked by facial and shoulder massage and relaxation exercises. Eye yoga strengthens the eye muscles and makes them more flexible; it relaxes the tissues and fascia around the eye and improves their relaxation and circulation. It also trains the binocular coordination. Though not yet studied systematically, ancient reports and modern clinical observations show that eye yoga can have a positive effect on myopia, hyperopia, and oculomotor problems. Our patients with low vision, e.g., suffering glaucoma or optic nerve damage, regularly report that eye yoga relaxes their eyes and feels good for their vision.

To understand how coordinated eye movements are important for normal vision, imagine a dancing couple moving effortlessly to the music with their arms, legs, and body in a perfectly coordinated manner. But if you or the partners pulls too hard, or if the other is too stiff or inexperienced, then the immaculate beauty of the flawless dance vanishes. In a similar fashion, our “dancing” eyes have to perform in a coordinated way throughout the day.^21^ Even during sleep, fast eye movements take place, especially when dreaming. Eye movement, like breathing or the heartbeat, is a 24-hour nonstop affair. Keeping the eye muscles in good shape is therefore essential for good vision. In addition to the strength and flexibility of the eye muscles, eye yoga promotes also the relaxation of facial muscles around the eyeball and may even improve blood flow.

Understanding your Vision Loss and Its Impact on Quality of Life

This part of the treatment is a kind of educational module with concrete and practical tips so patients can better understand their own visual field loss. The goal here is to teach the patients how to interpret the size and position of the scotoma and how it affects everyday activities. Also, patients need to know what other kinds of professions can offer further services (such as vision rehabilitation specialists and mobility trainers). This includes recommendations for dietary supplements to increase blood circulation and promote the health and flexibility of blood vessels and exercises to strengthen the cardiovascular system.

Vision Training

Depending on the type of visual impairment, home-based programs can be used, such as eccentric attention training and vision restoration training. We have previously developed several vision restoration training programs for home use which, depending on the type of visual field loss, can be used in conjunction with other procedures described here.^22-29^

Relaxation Techniques

Chronic stress is a risk factor in the development of vision loss, including the development of abnormal IOP.^30^ Especially in glaucoma, stress hormones in the blood (cortisol, endothelin) can have devastating effects on endothelial cells, leading to “vascular dysregulation.” Stress affects people who are constantly exposed to subtle but continuous levels of stress. Such stress burden is frequently found in people who have the desire to be “good” to others. They spend a lot of energy to take care of other peoples problems (such as family, friends, or coworkers) while neglecting their own needs. People with the following characteristics are particularly susceptible to vascular dysregulation (= stress susceptibility): those that are rather sporty and slim, ambitious, tendency to perfectionism with high expectations of others and of themselves, inclination to worry a lot, difficulties falling asleep or not being able to sleep through the night, high sensitivity to drugs, and those particularly sensitive to the sense of smell. This collection of characteristics is termed “Flammer syndrome,” which affects more women (70%) then men.

In summary, stress is one major cause underlying the development of vision loss, which is why it is important to regularly practice relaxation exercises to reduce such high and continuous levels of mental stress. Consequently, a holistic approach to vision therapy should include different relaxation techniques (meditation, autogenic training, progressive muscle relaxation, etc.) with the aim to reduce anxiety and depression to counteract the toxic effects of blood vessel-damaging stress hormones.^31-39^

Psychological Counseling

On the one hand, stress associated with visual impairment due to practical, psychological, and social problems is a consequence of low vision which includes anxiety, depression, and social isolation.^34,40-43^ On the other hand, life’s continuous stress can also be a major cause of visual impairment. Therefore, stress reduction is a crucial element of our therapy sessions. In addition to ACS therapy and eye yoga and relaxation exercises, psychological counseling of patients is another element of holistic low-vision treatment. During counseling sessions, different psychological aspects are discussed, such as coping with the vision loss or a negative prognosis and life perspectives. Since mental stress plays an important role in the onset and progression of visual impairment, the reduction of anxiety, the acceptance of the disease and attaining an optimistic attitude toward life through a brief session of cognitive psychotherapy can be very helpful and motivating to the patients. The goal of psychological counseling is for patients to adopt a new life perspective.^27,34^ In our practice, we teach our patients the following attitudes:

“Do not focus on what you have lost, strengthen what you still have!”

Goals of psychological counseling include the following:

- Acceptance of the disease and a positive attitude toward the new situation (coping);
- Reduction of anxiety through cognitive restructuring and strengthening of mental resources;
- Better handling of stress and worries;
- Promoting positive thinking by replacing negative thoughts with positive thoughts;
- Understanding, appreciating, and changing the burden on relatives and the social environment;
- Understanding the practical consequences of vision loss.

## THE CASE OF THOMAS P

To illustrate the power of holistic therapy for the treatment of low vision, let us consider the following case (Fig. 2). Thomas P, a 27-year-old economics student, was brutally attacked by three men in the early morning hours on his way home from a party. Thomas could not remember anything about the event when he woke up from a 10-day coma in the intensive care unit. Massive facial injury and brain damage caused one-half of the body to be partially paralyzed, and his vision was severely affected in both eyes: he could not see anything on the left and very little on the right eye. Through neurological rehabilitation, his ability to move recovered rather well and even his vision recovered spontaneously in the right that eye to 80%. The left eye, however, remained almost blind with 10% vision left.

About a year after this tragic event, Thomas came to us with the desire to regain a little bit of visual acuity so that reading would not be so difficult. Given his traumatic experience of the terror attack and his serious (90%) vision loss on the left eye, Thomas had a surprisingly positive attitude. His positive nature immediately earned him the sympathy of our entire team and fellow patients. He laughed a lot. He even had a certain self-irony when talking about his traumatic experience; in short, he had well accepted his vision loss. He did not resist his fate or mourned about the loss of his life before losing his vision. He rather wanted to take matters into his own hands and had a clear plan for his future.

In Magdeburg, Thomas was treated daily for 2 weeks with ACS, he was psychologically counseled, and he learned eye yoga and different relaxation techniques. In his spare time, he followed our recommendation to have more fun in life. At the end of the treatment, his visual field index increased from 10 to 15%, which he found to be a very satisfying experience because it was a big step in the right direction. Subjectively, he noticed that he could better see neon signs with his left eye, that everything was “somehow brighter,” and that he had the distinct impression that his brain was working more effectively. He said “I am satisfied; but more recovery would have been better, of course.” We pointed out that the ACS therapy effects might further improve in the following weeks if he diligently continued regular eye yoga, vision training, and relaxation exercises, as this would definitely increase the chance for further recovery.

Thomas was very ambitious and did just that: the “full workout program” daily, for months. When Thomas returned for a follow-up 4 months later, his improvements were remarkable: his visual field had improved from 15 to 74%!

He was very happy with the vision improvements. With a text message sent from his smartphone, he wrote:

“... the visual field improvement is really incredible. I see more, even very little things. I already have tears in my eyes because of happiness. So it is really incredible. And the treatment has made my left eye-sight much better. I am doing eye yoga every day now and I am much happier. Now I know, I did not lose an eye, but I won a healthy one....”

Thomas is a rare case as only few patients experience such dramatic recovery. But not all patients are as young and optimistic, and many are not as diligent with their vision homework. Nevertheless, It is for cases such as Thomas, improvements of vision in everyday life are a real incentive to continue studying the holistic approach to vision restoration, despite the skeptical attitude of many professionals toward treatment other than IOP reduction.

## CONCLUSION

The traditional paradigm “blind stays blind” is too pessimistic. It is not true that vision recovery is impossible. Rather, there are many good reasons for a more optimistic outlook with more light at the end of the tunnel. We are just at the beginning of a new journey of the science of low vision and its rehabilitation and restoration. For further information contact: info@savir-center.com or go to: www.savir-center.com
